# Health science staff and student experiences of teaching and assessing clinical skills using digital tools: a qualitative study

**DOI:** 10.1080/07853890.2023.2256656

**Published:** 2023-09-19

**Authors:** Annie O’Brien, Cuisle Forde

**Affiliations:** Discipline of Physiotherapy, School of Medicine, Trinity College Dublin, Dublin, Ireland

**Keywords:** Clinical skills, remote, competency, qualitative, digital tools, medical education

## Abstract

**Introduction:**

Once considered a supplement to traditional teaching approaches, digital tools now play a pivotal role in building core clinical competencies. This study aims to explore staff and student experiences of navigating the challenges of teaching and assessing clinical skills using digital technology. It also aims to provide insight into what skills, or aspects of skills, may be best suited to digitally enhanced teaching, thereby advancing the future of health science education.

**Methods:**

This qualitative study comprises the second phase of data generation for a mixed-methods research project entitled DEPTH (Digitally Enhanced Practical Teaching in Health Science). Health science staff and students expressed interest in taking part in the current study during the first stage of data collection. Qualitative data was collected in January 2022 through semi-structured group interviews and individual semi-structured interviews. An interpretivist qualitative research design underpinned by a critical realist epistemological position was used. Themes were generated following Braun and Clarke’s 6-step process for reflexive thematic analysis.

**Results:**

Overall, 10 staff and 8 students across 11 health science disciplines participated in this research. Fourteen hours of transcripts were analysed and 4 themes generated. Our findings highlight the suitability of digitally enhanced teaching for low-stake skills requiring visual and auditory training, while skills requiring tactile training require in-person practice to build student competency. Importantly, our findings indicate a desire for increased remote teaching. While our work was not specifically aimed at documenting experiences related to the Covid-19 pandemic, all participants had lived experience teaching or learning during the pandemic and many spoke specifically about this.

**Conclusions:**

The timing of this paper captures a novel moment in the history of clinical pedagogy. Staff and students advocate for the continued integration of technology into health science education generally, and clinical skills teaching specifically. For this to be successful, judicious selection of methods, skills, skill components and technology, that can be appropriately mapped onto specific learning outcomes, is required.

## KEY MESSAGES

1. Staff and students expressed a desire for more hybrid teaching of clinical skills but are sceptical about the suitability of skills, or aspects of skills, for remote teaching.
2. Clinical skills requiring auditory and visual training can effectively be taught using digital tools while skills requiring haptic awareness and manual dexterity necessitate in-person practice to consolidate learning.
3. The findings from this paper informed the development of an online, open-access, educational resource to support staff and students in maximising technology to support practical skill acquisition.

## Introduction

Technology supports a variety of flexible teaching approaches [1] that are ‘on-line and off-line, on-site and off-site, synchronous and a-synchronous, formal and informal, vocational and recreational and more’ [2]. Although technology has become a well-established tool for teaching clinical skills in third-level education (i.e. higher level institutes, universities and colleges) [3–5], technology has traditionally been considered a supplement to traditional classroom-based learning rather than an alternative [6]. Digital tools became essential in facilitating remote learning at the onset of the Covid-19 pandemic, and they supported the teaching of core clinical competencies required for completion and accreditation of health science degrees. This included soft skills (i.e. cognitive, behavioural and affective competencies that enable one to work well with others e.g. problem-solving, perspective taking, communicating) and hard skills (i.e. objective and quantifiable competencies that demonstrate technical skill, gained through training, experience or practice e.g. patient assessment and treatment, laboratory tests).

The pivot to remote practical skills teaching presented challenges for those who teach and those who learn [7]. Previous research, much of which was conducted in the early stages of the Covid-19 pandemic, revealed that students in the health sciences felt disadvantaged by remote clinical skills teaching and missed in-person practice [8,9], which they believed was critical for building skill competency [10,11]. Approximately 42% of students believed practical skills were not suited to distant learning, while 75% felt practical skills should be taught in clinical environments [6]. Furthermore, almost 75% students reported not feeling confident practicing the skills they learnt remotely in a clinical setting [6]. Indeed, some educators deemed distant teaching of practical skills an inadequate substitute for in-person learning [12].

Although research into attitudes towards online learning increased at the onset of the pandemic [6,11,13–15], there remains a dearth of literature on the experiences of overcoming the challenges to acquiring and assessing clinical skills using digital technology. While there is evidence that digital tools successfully support the teaching of some soft skills [16–19] less is known about whether clinical skills, or aspects of skills, are suited to distant learning or what form of technology is best suited to practical clinical skill acquisition [7]. For example, educators have expressed concerns around the feasibility of developing technical skills (e.g. setting up and troubleshooting experiments) in virtual labs [20] and collecting a holistic patient history virtually using a webcam in the absence of non-verbal communication [8]. However, much of the research to date on attitudes towards the acquisition of clinical skills using technology has been collected using cross-sectional survey designs [9,11,13,14,21]. This method, while informative, limits the ability to explore factors influencing participant beliefs and the evolution of attitudes as universities learn to adapt to new teaching methods. They are also limited in their ability to explore in depth why skills, or aspects of skills, were perceived as unsuitable for remote learning [7,22].

Previous research findings indicating that online education is viewed negatively by students may be associated with the treatment of online education as a substitute to traditional education and perhaps consequently considered an inferior teaching method. In the post pandemic era, students and staff are now largely in favour of a blended teaching approach [23,24]. Within the health sciences, whether to – and if so, how best to - support blended learning for practical skill acquiral is an understudied area of education research [7]. This research gap, coupled with negative attitudes towards remote practical teaching during the pandemic, may lead those who teach and those who learn to disregard the potential educational value of technology in this area. A necessary step to inform the future of health science education is to therefore learn from the experience of staff and students who have taught and learned clinical skills with the help of digital technologies.

## Objectives

Recent years have seen educators revisiting and redeveloping their approaches to practical skill teaching [20]. As they harness their learnings from the pandemic years, new technologies become available and various pedagogies are trialled. This study primarily aims to explore the experiences of university students and educators when navigating the teaching and assessment of core clinical competencies using digital technology and understand how this teaching effects students’ learning experience. A secondary objective is to identify the skills, and components of skills, suited to distance or digitally enhanced teaching.

## Method

### Design

The current study is the second phase of data generation for a mixed-methods research project entitled DEPTH (Digitally Enhanced Practical Teaching in Health Science).

Qualitative research facilitates an in-depth investigation of a phenomenon through exploration and consideration of multiple perspectives [25]. Thus, we used an interpretivist qualitative research design underpinned by a critical realist epistemological position that encourages listening and learning from various lived experiences to understand a phenomenon. Semi-structured interviews were used to generate data. Transparency in reporting was ensured using standards for reporting qualitative research checklist [26] (see supplementary materials). Ethical approval was granted by Trinity College Dublin’s School of Medicine Research Ethics Committee [Application Number: 20210604].

This research benefited from public and patient involvement (PPI) in the form of a student advisory group (SAG). A call for health science student to volunteer with a SAG for the DEPTH project took place in September 2022; a promotional flyer was shared via email with class representatives, on social media platforms (e.g. Instagram, Twitter), and physically posted in health science faculty buildings. The SAG comprised of 12 1^st^-3^rd^-year health science student volunteers, from a range of health science disciplines (e.g. medicine, nursing, pharmacy). Consistent with our methodological approach, the SAG was involved at all stages of this work in an advisory capacity. Their feedback was used to inform and consolidate the authors’ work as the project progressed.

### Sample

The first phase of this research project consisted of an online survey for health science staff (i.e. educators) and students. Phase one recruitment for the online survey involved distributing a promotional flyer advertising the study on the social media platform Twitter. Emails were also sent to staff and students in the Faculty of Health Sciences in Trinity College Dublin. Staff and students were eligible to take part in the current study if they (1) completed the online survey comprising phase one of the project; (2) taught or studied a health science subject, and (3) were based in a third level institution, in Ireland or abroad.

When completing this survey, participants were invited to provide their email address in an open-text box if they were interested in taking part in phase two (i.e. the current study involving interviews). Of the 60 survey participants that expressed an interest in phase two, 30 were invited to take part using non-probability purposive sampling. This method of sampling was used to ensure a representative sample of health science disciplines and to facilitate discussion among multiple perspectives, enriching data collected and reducing likelihood of bias.

Of the 30 people contacted via email with a participant information leaflet and consent form, written consent was received from 18 participants (10 staff, 8 students). Participants were affiliated with a range of third level institutes across Ireland and Australia.

It’s important to note that the timing of this study is such that those invited to interviews would have experienced teaching and learning during pandemic related restrictions, which we expected to provide rich experiences from which we could learn how to support future teaching. While this research aimed to gain information on participant’s experiences of a topic (i.e. digitally supported practical teaching in the health sciences), experiences were not limited to a specific period in time (i.e. during the pandemic). While it was expected that participants would draw on their experience gained during the pandemic, we welcomed participants with experiences gained before and after the Covid-19 pandemic.

### Procedure

**Figure 1.**
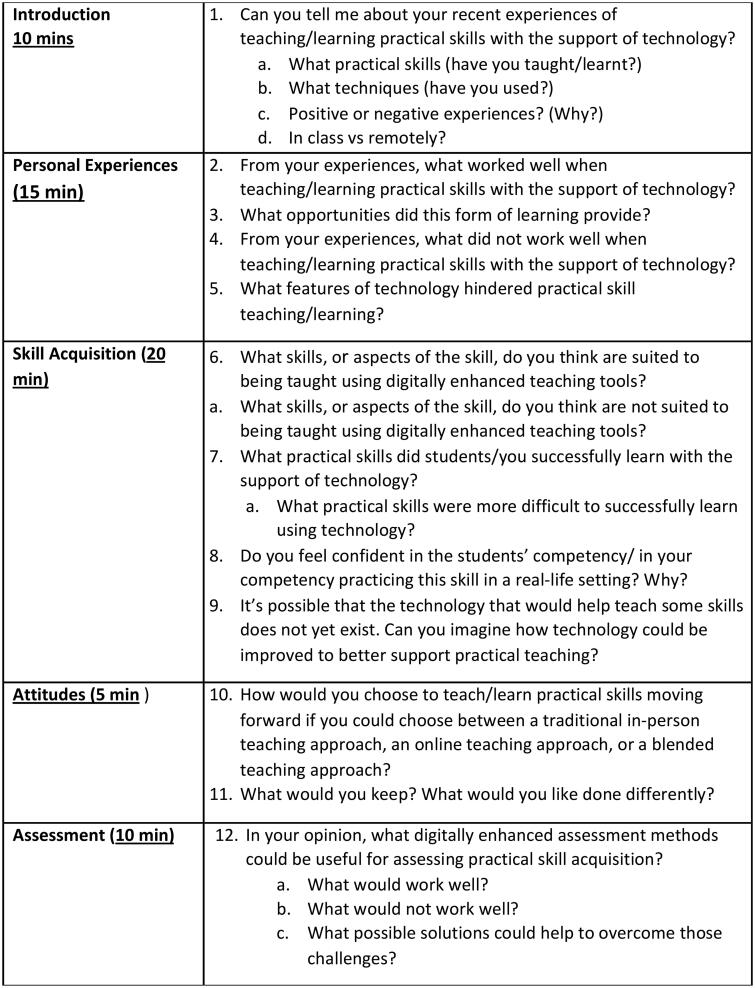
Interview guide used.

A previously published literature review [7] from the DEPTH project informed the development of an interview guide (see Figure 1). The SAG piloted the interview guide prior to data collection and minor edits were made accordingly. The interview guide comprised of open-ended questions. Additional prompts and exploratory questions were used at the discretion of the interviewer to clarify detail and encourage further elaboration of topics.

Where participant availability did not align with that of other participants, the option to take part *via* a semi-structured individual interview was provided, otherwise group interviews were used. This provided flexibility while also supporting the methodological approach given that individual interviews would provide in-depth perspectives while group interviews would facilitate an interactive exchange of diverse opinions and experiences [27]. Student and staff group interviews were conducted separately to provide a safe and non-confrontation space for disclosure. Two staff group interviews, comprising three participants each, and two staff individual interviews were carried our lasting 60 – 90 min each. Due to conflicting student schedules during a busy time in the academic year, one group interview (*n* = 2), and six individual student interviews were carried out, lasting 60 min each. The five items underpinning information power in qualitative research [28] were considered in relation to data saturation, and both authors collaboratively agreed data saturation was reached by the final staff and student interviews when no new codes were generated [29].

Interviews were audio-recorded with participants’ verbal consent using a dictaphone. Audio-recordings were immediately uploaded to a secure online storage system, where they will be stored securely for 7 years. Original data was deleted from the recording device. Interviews were transcribed verbatim with identifiable information redacted. Participant pseudonyms were created to ensure participant confidentiality.

As this work requires researchers to remain reflexive and to consider how their background and experience informs their perspectives the following personal detail is provided on the authors: AOB conducted the interviews online *via* Zoom between December 2021 - January 2022. AOB is a research assistant with an MSc in Clinical Health Psychology and experience in qualitative research methods. CF is the principal investigator and developed the protocol for DEPTH. CF is a physiotherapist with a full-time academic position. CF runs a postgraduate online course for clinicians and teaches practical skills with support from digital technology. During the course of study development and data generation, the authors continuously reflected on their positions and perspectives, considering how these evolved and interacted over time, with an acute awareness of how such perspectives might influence the research process.

### Analysis

Data analysis followed Braun and Clarke’s 6-step iterative process of reflexive thematic analysis (TA) [30] using software package NVivo 12 (QSR International, Australia). In line with our critical realist positioning, an inductive approach to reflexive TA was used as it is ‘‘a theoretically flexible method’’ [30]. This approach enabled us to capture the breadth of experiences across a diverse sample, ranging in location of study (i.e. country), discipline and year group. This bottom-up approach, which used open questions and prompts, enabled participants to share experiences that were personally meaningful to them, without being limited by theoretical pre-conceptions that may arise from a top-down approach to data generation [31]. This approach has also been used in other qualitative research of digitally enhanced learning [32,33].

**Figure 2.**
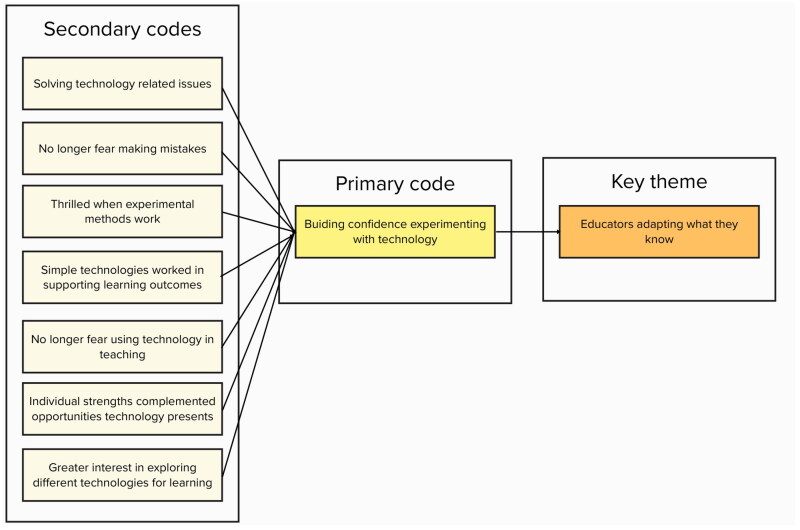
Example of the mapping process between codes and theme.

The six steps are as follows: firstly, both authors engaged in data familiarisation by reading and re-reading transcripts and noting down early impressions. Secondly, both authors independently generated initial codes by meaningfully and systematically assigning meaning to the data. Codes were discussed between researchers through investigator triangulation to ensure credibility of final codes [34]. Thirdly, codes were collated into candidate themes. Next, before candidate themes could progress towards becoming themes, they were discussed, reflected upon and adjusted accordingly with a team of experts (*N* = 2) from the university’s Academic Practice, (i.e. employees of Academic Practice, with a background in online education, specifically employed to support academics in pedagogical research and practice). The team of experts tested the candidate themes for referential adequacy by returning to the raw data. This process of peer-debriefing (i.e. comparing notes on codes and candidate themes, discussing differences and reaching agreement on the final interpretation of meaning [35]) informed the refinement and naming of the final themes, carried out by the first author. An example of the mapping and interpretation of codes to themes is illustrated in Figure 2, inspired by Ritchie and Spencer’s framework analysis model [36]. Finally, appropriate quotes were selected for reporting and discussion of findings. The authors kept a reflective journal to use as a tool to explore thoughts and perceptions arising throughout data collection and analysis.

## Results

The final sample included 10 educators (representing 7 health science disciplines) and 8 students (across 3 year groups representing 6 health science disciplines). Participant characteristics are detailed in Table 1. Staff positions and experience varied from professional to academic staff at various levels (i.e. lecturer, professor etc). Details on the positions staff members held is not disclosed to protect anonymity.

**Table 1.** Participant group characteristics (*N* = 18).

Participant		n		n
	Student	8	Staff	10
Gender				
	Female	6		9
	Male	2		1
Discipline				
	Pharmacy	2		
	Nursing	1		
	Dentistry	1		1
	Physiotherapy	2		
	Radiation Therapy			2
	Medicine	1		
	Sport and Exercise Medicine	1		
	Human Nutrition and Dietetics			1
	Speech and Language Therapy			1
	Physiology			2
	Learning Technology			1
	Clinical Skills			2
Degree				
	Undergraduate	7		
	Postgraduate	1		
Year of Study				
	1st	2		
	2nd	3		
	3rd	3		
Mature students		3		

Note. A mature student is defined as a student aged 23-years or older on January 1st of the year of course commencement (37).

Four themes were generated using the process of reflexive TA. These themes are mapped onto the study research objectives and presented alongside their corresponding codes in Table 2.

**Table 2.** Themes and corresponding codes generated using reflexive thematic analysis.

Research objective	Theme	Code	Example quote
1 Explore the experiences of university students and educators when navigating the teaching and assessment of core clinical competencies remotely, and how teaching effects learning.	Educators adapting what they know	Difficulty adapting	*The delivery of radiation, there’s a real practicality to that… How you replicate that when you can’t have that hands-on… that kind of gave us big headaches*. – Staff08
		Educators doing the best they can	*In the middle of the pandemic, it became very much ‘‘but do whatever you can’’ and every idea is a good idea when you have nothing.* – Staff06
		Building confidence experimenting with technology	*We become confident as well I think in going ‘‘oh, did you not try not try this? Or how about doing this?’’* – Staff03
		Demonstrations of innovation and creativity	*We have a Massive Online Open Course… for patients and the general public… we were able to use that with some of the clinical placement hours for students… it actually then became part of teaching even though we never set out for it to be like that in the first place.* – Staff05
		Judicious use of technology	*You shouldn’t use technology just because it exists out there in the market. You have to see whether it actually suits.* – Staff07
	How we teach practical skills will change	Passive to active learning	(*Students) preferred to go and look at the videos. So they like self-directed learning, which is good, very good, for long term learning and for lifelong learning… online learning encourages this.* –Staff07
		Future skill teaching should be blended	*I think blended learning is the way to go in the future*. – Student01, Nursing
		Imagining the possibilities	*If they could use the interactive patients, (not) like the finicky ones, but that speak and do all those weird and wonderful things…that replicate actual patients*. – Student07, Physiotherapy
	The integrity of online practical teaching and assessment	Pressure to uphold standard of teaching	*We’ve standards of proficiency that have to be met and you have to stand over that someone can physically do that job.* – Staff05
		Standardisation of competency assessment	*I think there’s a chance to bring standardizable formats in… (assessment) was always patient based, they wanted a live patient and I think we’re moving away from that for some exams.* – Staff01
		Integrity of online assessment	*I think it’s a big fear in our school in particular, about standing over online assessments.* – Staff05
2 Identify the skills, and components of skills, suited to distance or digitally enhanced teaching.	Layers of multifaceted skills can be taught online	Building competency in low stake skills	*They were signed off (as competent) by a clinician remotely - they were just floating on air. You know, they just thought yes, I can do this*. – Staff08
		Complex skills are learnt in clinical settings	*To learn the skill, they have to perform the skill. You can do all of the supporting theoretical teaching, the pre-recordings, the video … but if they don’t do it themselves, that’s it*. – Staff10
		Unsuitability of some skills	*It’s hard to just solely learn certain practical skills online.* – Student08, Pharmacy

### Research objective 1: Explore the experiences of university students and educators when navigating the teaching and assessment of core clinical competencies remotely, and how teaching effects learning

#### Theme 1. Educators adapting what they know

The rapid onset of the pandemic required educators to quickly adapt traditional teaching approaches for distance learning. Many educators were anxious that their knowledge of the technology available to them was ‘so low level’ (Staff03) that they would struggle to adapt to this new normal:

This took so much of our time, like the summer of 2020 for me was spent learning to move online. – Staff10

Training staff in using technologies ‘took a lot of effort in the first few months’ (Staff07). Staff described doing their best in a rapidly evolving situation and initially used familiar technologies to support students in achieving learning outcomes. In time, some educators wanted to further upskill, to understand and employ the elements of good design in their digital learning resources, but struggled to do so:

What I find frustrating is I have a picture of how good it could be and I don’t know how to make it because I don’t have the money or the time or the resources or the institutional support to actually do that. – Staff03

Many educators were surprised at their ability to swiftly and successfully adopt new digital tools into their teaching. It was unanimous among staff and student participants that technology should not be integrated into practical skill teaching solely because it is available, but to support specific learning outcomes in skill acquisition and competency. Introducing technology because it is ‘flashy and shiny and cool’ (Staff03) was described as having the potential to harm the learning experience:

If technology is going to be used, it needs to be productive… and not just there (because) you want to bring in technology. – Student01, Nursing

Overtime, educators identified their passion and commitment to teaching as a strength and began to tailor technology to complement their teaching approaches, rather than changing their teaching approach to suit the technology:

I think, having not grown up with technology, then you come up with solutions that are a blend. You’ve got a whole other set of experience that you can kind of weave into the technology, or vice versa. – Staff03

Staff became more confident that learning outcomes could be met using simple technologies, such as video-conferencing platforms and granting remote access to software through shared screen features. The realisation that staff could adopt a step-wise approach reduced their fear of using advanced technologies (e.g. simulations and virtual realities) to achieve learning outcomes:

It’s not about trying to push (teaching) forward into this very sci-fi zone, it’s just about really enhancing what we’re currently doing, and it’s that balance of the relatively low-tech approaches which can be really effective. – Staff02

### Theme 2. How we teach practical skills will change

Participants described experiencing a movement away from the ‘Socratic method’ (Student06, Medicine) of teaching, as students were required to take responsibility for their learning and use online materials to learn practical skills independently. Students advise ensuring open pathways of communication with teachers will improve opportunities for active learning:

I think especially now after the pandemic, they should try and do … a total rehaul or switching around of what’s online, what’s in-person… I think for most people, not only teaching wise, but what’s better for them, for their social life, their family life, is to give them a chance to do blended learning. – Student03, Pharmacy

Opportunities presented by digital technologies continue to grow as technology improves and further secures its place within the healthcare setting. Educators agree that practical skill teaching ‘should be incorporating’ (Staff06) technology and stay up-to-date with the latest advancements in medical education. It was unanimous among staff and students that blended learning will best support practical teaching moving forwards:

A blended approach will be exactly what we need. We need to learn from Covid … We’ve learned a lot of things I think that before we didn’t do, and now you say I am not going back to the old system… this is a better approach. – Staff10

To further build on the momentum gained over the past two years, educators are imagining new ways to use technology to enhance practical skill competency, and are considering how advanced technologies could enable application of multiple soft and hard skills concurrently:

We have something like SIM man which is used in clinical skills. They’re adapting the environment *around* that so when using virtual reality or augmented goggles, students can feel like they’re actually in the hospital or in the operating theatre. – Staff04

Gamification is very interesting for me. You have your virtual ward for the day, here’s your workload, and they have to prioritise which patient you go to next, which task do you do next… It’s an interesting concept to make it a bit fun, while it would also motivate them and address very serious things – critically thinking, prioritization, some of the procedural type skills. – Staff06

### Theme 3. The integrity of online practical teaching and assessment

The integrity of online assessment is described as a ‘major concern’ (Staff05) across health science disciplines, as there is a lack of evidence-based research informing standardisation of remote assessment practises. Educators felt individual responsibility to ensure online skill teaching was delivered to a high standard, despite an absence of online education strategies and procedures within their school. Some staff struggled with the pressure to uphold this high standard of teaching when the workload of designing digital resources went unrecognised and unappreciated both internally (i.e. by students, the institution) and externally (i.e. by the public):

In college it was one thing, but the general public, well, it was really a bit upsetting … Just to provide one hour of lecturing, it was like 6 hours of recording and editing and people did not realize that so they were like ‘oh we’re paying just to have people sit around in their place and do nothing’. – Staff09

However, some staff believed technology introduced standardisation of assessment methods, facilitating equity and fairness in exam procedures previously based on real-world patient presentation that could vary in complexity between individual student assessments:

We made standardised patients so that everybody got asked the same questions at the same time which is something we couldn’t do (with real patients). Now that Covid is over, we’re saying actually that works so well, and is a lot fairer. – Staff01

Both staff and students felt that online or recorded video demonstrations of skills were ‘on par’ (Staff06) to in-person exams. Students felt confident that approaches used (e.g. remote proctoring software) upheld written exam integrity as it ensured that ‘remote examinations aren’t just being completely skewed by everyone doing open book exams… it’s pretty much exactly the same as a regular in-person exam’ (Student07, Physiotherapy). Similarly, assessment of virtual OSCEs on videoconferencing platforms was described as ‘very similar to what you would do in-person’ (Student03, Pharmacy), while a recorded practical enabled students to demonstrate skill acquisition – ‘you have to perform it and you’re either going to get it or you’re not’ (Student02, Sport and Exercise Medicine).

Some laboratory-based skills could be assessed remotely by examining outcomes rather than procedures, for example pipetting:

When you do some protein assay, if the replicates are really close together you can tell the student has the pipetting skills acquired. – Staff09

Despite its suitability for assessing low-stake soft skills, participants agreed that remote assessments could not substitute the integrity of in-person assessment for high-stake skills. It was felt that in-person assessment was necessary to ensure competency in high-stake skills required for degree completion and accreditation.

### Research objective 2: Identify the skills, and components of skills, suited to distance or digitally enhanced teaching

#### Theme 4. Layers of multi-faceted skills can be taught online

Participants agreed that students often theoretically knew and technically performed individual skills learnt online very well. Core competencies were described as ‘multi layered’ and ‘multifaceted’; participants described the teaching and learning of layers of skills, or low-stake skills (e.g. communication and history taking), as appropriate for online environments:

I suppose all the pieces of the puzzle, in terms of the individual skills that make-up that big piece of handling a case load and managing that working environment, I think you can go after the overwhelming majority of those individuals skills with the use of, or help of, technology. – Staff02

Ideally an OSCE (Objective Structured Clinical Examination) should be in person, but there are plenty of stations that could be adapted and you could have a virtual OSCE. – Staff04

Students noted that there was a need for distinct tailoring when teaching skills that are applied to both in-person or online interactions:

We’re all taught how to communicate with someone face-to-face… there’s definitely a difference between how you communicate with others in-person and online, and you have to allow for things such as the lag in response between your voice and your body language, people’s reactions… – Student08, Pharmacy

However, most participants reflected that students struggled when applying individual skills they learnt online to real-life scenarios that required application of multiple skills simultaneously. This was thought to result from the holistic application of individual skills, for example the ‘clinical reasoning…the reading between the lines, reading the nonverbals, and putting all of that together’ (Staff03). Similarly, when teaching or assessing remotely, some staff struggled to facilitate the acquisition of soft skills in public speaking and teamwork. Educators therefore agreed that technology ‘complements’ traditional teaching methods (Staff06).

Clinical skills requiring visual and auditory feedback could be effectively taught using visual and auditory resources. Students felt confident performing ketone analysis and auscultations after watching and listening to recorded online materials, and could build competency in patient treatment through videoconferencing platforms:

When you’re listening to heart sounds or trying to locate the lugs or pleura, things like that, I feel it was nearly easier using online resources rather than in-person practicing because it’s all very specific. – Student07, Physiotherapy

We hooked the student up with a clinician via Zoom so that the student would create their dosimetry that they would normally create clinically or in the classroom… the student shared their screen and talked them through the plan they had produced, and then were assessed and deemed competent digitally. – Staff08

Students struggled however to build confidence in their knowledge of skills that were dependent on haptic awareness and manual dexterity. Haptic training was perceived as particularly difficulty as these skills required familiarity with equipment, haptic and expert feedback and in-person practice. The opportunity to practice tactile skills in an educational environment builds student confidence in performing the skill in a clinical or laboratory setting:

There’s a mechanical memory of your hands when you’re doing this action that you don’t necessarily get when you’re at home watching on a laptop. I can do my best, I can get similar instruments and try and practice. But something about using hospital instruments and then a tissue or a practice pad, there’s definitely a difference, and you can tell. – Student06, Medicine

I’ve also tried some sort of software that is supposed to be helping in teaching wet lab skills, it’s teaching the protocol, but it’s not teaching the skill itself… it can teach you the order of the steps that you have to do… What is also important, I think when you do wet lab is to minimize spills, risks and things like that. So if you are not actually doing the thing, you cannot learn how to prevent that virtually. – Staff09

The importance of in-person practice was emphasised by all participants, who agreed that to become familiar with manual techniques, and navigate the range of potential outcomes that arise in a clinical or laboratory setting, in-person practice is essential.

## Discussion

In response to an increased exposure to technology enhanced teaching, this study aimed to explore the experiences of key stakeholders (i.e. staff and students) towards digitally enhanced practical teaching and assessment, and identify components of clinical skills suited to distance or digitally enhanced teaching.

This study aptured both positive and negative experiences of digitally enhanced teaching and assessment, which is in line with the results of other similar studies [38–41].Overall, those who teach and those who learn were consistent in voicing a desire for practical skill acquisition to be taught using a approach. This is not surprising and aligns with the universal design of learning which promotes greater flexibility and multiple means of engagement, representation and expression [42] which can be achieved using a blended learning approach in health science education [43]. The more pertinent question this research addresses is what we can learn from the experiences of our participants to inform future curricula.

### Experiences of digitally enhanced teaching and assessment

Educators were vulnerable in their admission of feeling extrinsic and intrinsic pressure to maintain high standards of teaching during the acceleration of technology enhanced teaching. This pressure is recognised as coming from university culture, evolving student demands and public expectations [44], and from oneself. There is increased responsibility on educators to continuously upskill and to demonstrate competency in designing and developing digital resources to achieve learning outcomes, leading to greater contributions of personal time and effort in maintaining high quality teaching. Despite this, participants felt discouraged to uphold this high standard when their institutions predominantly recognise and reward academic staff for their researcher output [45,46].

**Figure 3.**
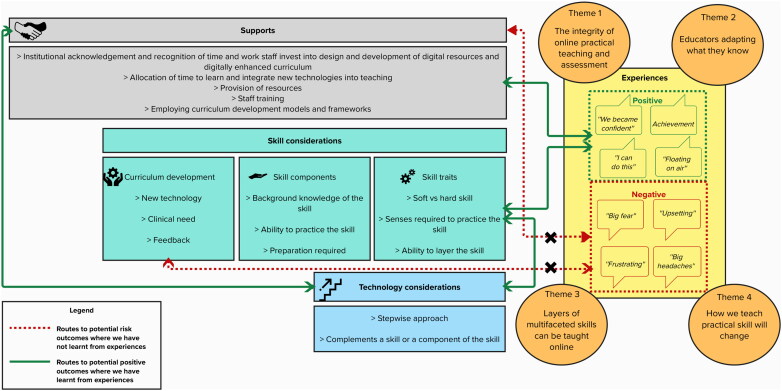
Infographic illustrating main study results and the interplay between variables, experiences and processed as described by participants. *Note*. Themes are presented as the four cornerstones to experience.

Those who teach and learn openly shared their experience of working during the pandemic as a period when they felt panic, frustration, monotony, undervalued and even resentment, however as time passed several positive experiences emerged leading to feelings of relief, hope and opportunity. Going forward, adapting digital technology into practical teaching can take a more measured or stepped approach, and leverage lessons from this experience. Through shining a light on the perspectives of those with lived experience, this research also highlights the risks of poorly integrated digital technology into health science education. We have graphically displayed our results including the interplay between experience, feelings expressed and tangible variables mentioned by our participants in Figure 3.

Educators spoke of their hesitancy in integrating advanced technology into their teaching methods. This lack of self-efficacy and experience using advanced tools can be explained by Regmi & Jones’ theoretical framework [22], which identified poor digital skills as a teacher-level barrier to effective online learning. Lack of digital skills and self-efficacy has continuously been reported as a major barrier to the successful adoption of digitally enhanced teaching [7,8]; indeed, an inverse relationship between negative attitudes towards digital tools and predicted proficiency has been reported [47]. However, our findings recognise the resilience, adaptation and innovation educators demonstrated during remote teaching. As they became more comfortable and confident with basic technology, they were motivated to further build a repertoire of digital skills and continue successfully incorporating technology into their clinical skills teaching. To do this successfully, educators felt it necessary to have institutional support, and to take a step-wise approach to technological integration.

The risks associated with poorly considered use of technology in practical teaching were clearly expressed by participants as part of this work. Feeling undervalued and frustration were experienced when technology was poorly incorporated into teaching, highlighting a need to consider curriculum development models and incorporate student feedback into teaching practices. Our findings suggest that in the post pandemic era this involves a stepped process whereby educators initially consider whether the learning outcomes can be better facilitated using digital tools, they then introduce simple and familiar technologies (e.g. video-conferencing platforms, remote access to software) to support learning outcomes before incorporating more advanced technologies (e.g. simulation) if and when a teacher feels confident in doing so, and if it will further consolidate learning. As academics incorporate new teaching supports into their work, both institutions and students can support this process by recognising small changes towards better practices rather than expecting sudden changes to high-tech solutions. This stepped approach is represented in ‘Technology considerations’ in Figure 3. As depicted using double sided arrow, there is an interplay between skill considerations, support and technological considerations, however technological considerations, while playing a central role, are presented in Figure 3 as somewhat removed from other subject specific considerations. This central, yet slightly removed representation reflects staff and students’ concern that there needs to be a good ‘fit’ between a technology chosen to teach a skill and the skill or component of the skill itself.

As represented under ‘Supports’ in Figure 3, educators achieve a step-wise process of technology integration by following a curriculum development model; an example of this is demonstrated by Khamis and colleagues [16] who developed a methodological step-wise model for simulation curriculum development in clinical skills. Their approach was informed by Kern and colleagues’ 6-step approach to medical education curriculum design, principles of simulation design in medical education, and expert feedback [48]. This model details the steps necessary for curriculum development (e.g. identify broad learning outcomes and measurable objectives; individual assessment and feedback) and how they can be mapped concurrently with simulation development. Using a step-wise approach to successfully integrate technology into health science education should be incorporated into school strategies, alongside the recommended theoretical models for curriculum development across a variety of digital tools. Universities should simultaneously provide and promote digital skills training among staff to support them in adopting suitable technologies.

### Skill components suited to digitally enhanced learning

Alongside exploring and describing participant experiences of digitally enhanced practical teaching, this research aims to identify the components of clinical skills that are suited to digitally enhance and/or distant learning. Perhaps the most important finding from this research is that not only did staff and students describe adapting quickly to digitally enhanced skill teaching, they found it successfully facilitated the acquisition of low-stake skills. This qualitative finding is supported by meta-analyses of experimental research designs [5,18]. Indeed, a Cochrane review found patient simulations can improve student skill acquisition (of both soft and hard skills) over traditional means and are a teaching tool students enjoy interacting with [18].

Thus, this paper further advances knowledge gained from quantitative research by enhancing understanding of why some skills may be more suited to digitally enhanced teaching. Participants reported that digital tools facilitate competency in practical skills requiring visual and auditory training, often taught with the support of videos, online demonstrations and simulations. Students can gain exposure to sounds or images needed in their training through an array of digital resources, however consolidation of competencies requiring tactile sensations or haptic awareness may be more difficult without physical practice and feedback. To put this finding in context, an example of a skill requiring auditory training is auscultation. Some skills involving haptic training are palpation, passive movement of joints, locating bony landmarks, assessing skin texture and obtaining a pulse. Visual training is required to read medical images, assess gait, and to analyse biological materials.

Learning a skill is often described as a layering process, as the adage ‘see one, do one, teach one’ describes. In our work students and staff described the use of technology as having the potential to improve two of these layers with staff taking time to ensure pre-recorded demonstrations were of a high quality (‘see one’), and students being afforded the ability to make mistakes during simulations as they practiced skills (‘do one’). With a blended approach, educators and students may consider the layers that exist in the process of learning a skill and whether technology can be used to enhance one or more of those layers.

It is clear that educators want to incorporate what they’ve learnt over the course of the pandemic to continue improving future practical skill teaching. This also involves acknowledging and rejecting unsuccessful approaches. Despite the prevalence of negative attitudes towards online or distant skills teaching captured by cross-sectional studies and reviews [15,22], an important finding of this qualitative paper is that students welcome hybrid or blended education with open arms. This is of particular importance as clinical skills teaching has traditionally been perceived as unsuitable for digitally enhanced teaching. This paper thus advances the evidence base around digitally enhanced clinical skills teaching and skill suitability for remote teaching. This paper also highlights the risk associated with poor use of technology in practical teaching and details processes which can help avoid or reduce such risks. This paper contributes a new insight as it indicates that moving forwards, clinical teaching can continue to evolve to incorporate digital tools and enhance the learning experience in ways previously unimagined pre-Covid-19. Specifically, teachers are considering different ways to incorporate digital tools into the learning environment and bring enjoyment to the learning experience (e.g. Staff06 shares a desire to explore gamification and Staff04 mentions creating virtual realities of clinical environments). Learning is thus moving away from traditional methods towards something new and exciting; perhaps advancements would not have been so quick or readily accepted had the pandemic not happened.

The timing of this paper therefore captures a novel moment in the history of clinical pedagogy. The stepwise approach under ‘technological considerations’ depicts how initially using more familiar technological solutions to reach learning needs can lead to the eventual adaptation of more suited or perhaps more complex technological supports. This paper captures the experiences of staff and students at an exciting moment where the integration of technology into practical teaching is moving from basic to more advanced in response to student feedback and parallel change in the delivery of health care. Finally those who teach are encouraged to examine aspects of the skills they teach, in particular what senses are required, as well as whether aspects of that learning can be layered and certain layers supported by technology, as an important variable in the decision to use digital technology to support learning.

### Strengths and limitations

There are strengths to this research study that support its advancement of knowledge on this topic. This research is required to help inform and ground future curriculum development and a strength of our work is that it was performed at a time where reflection was possible, after the Covid-19 pandemic. Qualitative research exploring these experiences will enable those who design, develop and teach to effectively navigate alternative teaching approaches and use evidence-informed research when selecting appropriate methods to support practical skill teaching.

The researchers ensured rigor throughout each stage of data-collection and analysis through peer-debriefing with a team of experts and use of reflective journals. Quota non-probability sampling was employed to target participants (e.g. mature students) to ensure a representation of the student body, combat bias and to explore a range of perspectives across many health science disciplines. Another key strength of this paper is that the findings informed the development of an online, educational resource (OER). This OER is an online, open-access, evidence-informed tool that guides educators in selecting appropriate digital tools for soft and hard skill acquisition. It facilitates wider reach and impact of these research findings and can be accessed on the National Forum’s National Resource Hub, under ‘DEPTH’ [49] after registration with the platform, which is free of cost.

As part of our interpretation, we have developed an infographic outlining the main findings of our study and how they can be used to support student success (Figure 3). This infographic is not intended to function as a guide to developing or adapting course material, but rather to communicate our findings in a concise manner, displaying the numerous considerations in the area of technology supported practical teaching.

Study limitations are that many participants were affiliated with one Irish university, which may have impacted findings, and that naturally, all participant responses referenced the Covid-19 pandemic as this period was when much experience in digitally enhanced practical teaching was gained. Despite this, this paper focuses on utilising this period as a building block to enhance and advance clinical teaching moving forwards. Future research that aims to explore the use of a single digital tool to support the acquisition of a specific clinical skill may benefit from applying a theoretical model (e.g. The Dreyfus Model of Skill Acquisition) to understand how use of a digital tool supports a learner’s progression from novice towards skill mastery. While this model is a useful and appropriate model for describing competency progression, employing a similar model would not have been suitable for our study which explored perceptions of digital tools generally, and their use supporting the acquisition of a plethora of clinical skills across multiple health science disciplines.

## Conclusion

This research recognises that digital education has an important place in health science education, with a strong consensus among stakeholders that blended skill teaching is the way forward. Staff and students agree that the pandemic will positively impact future practical skill learning, as layers of multifaceted skills have successfully been adapted for remote teaching and there is an increase in technology use both in the classroom and clinical teaching sites. Educators however feel extrinsic pressure to support students in achieving core competencies without acknowledgement or consideration of the time required to learn and maximise new technologies. The risks of poor use of technology in practical teaching include staff and student experiences of frustration and feeling undervalued. Experiences of hope, opportunity and excitement were also expressed with clear potential to support student success through enriching teaching with digital technology. Thus, to maintain and advance the progress of digital education, previous experience teaches us that there is a need for support in maximising digital tools and the opportunities they present;there is also a need to consider several skill traits and technology traits.

Our results concur with previous research stating that there is a place for technology in practical health sciences teaching [50–52], however in learning from the experience of staff and students, we add both a note of caution and of anticipatory excitement going forward. We illustrate the main considerations in this area, as well as the important interplay between them. We propose that considering the role of technology in practical teaching demands iterative stepped and multifactorial considerations. Our work gives a platform to student voices who are expressing a desire for teaching practices to mirror technological advances that they see in health care settings and use in their daily lives, while also hearing staff who express concerns about maintaining high levels of academic integrity in their work while also demonstrating innovation and creativity in advancing their pedagogical practices with the support of technology.

## Supplementary Material

Supplemental Material

## Data Availability

A completed Standards for Reporting Qualitative Research (SRQR) checklist is available in the supplementary materials. Participants did not consent to having their data shared on an open repository.
