# Supplementary material for: Health science staff and student experiences of teaching and assessing clinical skills using digital tools: a qualitative study
Source: Ann Med. 2023 Sep 19;55(2):2256656. doi: 10.1080/07853890.2023.2256656 (PMC10512752; doi:10.1080/07853890.2023.2256656)
Supplement: Supplemental Material [file IANN_A_2256656_SM4879.zip › Supplementary Material.docx]

Supplementary materials

An extract from the lead author’s reflective journal

Data collection day #1

*‘’I think being a student during the Covid-19 pandemic, studying and learning remotely, has helped me to understand the challenges students faced over the past number of years…*

*Similarly, building up strong relationships with my educators who were teaching remotely, enabled me to bring compassion and understanding to this focus group with a number of staff members.*

*I remember during my MSc some students were impatient and unkind towards the challenges our educators faced as they attempted to adapt to the challenges of the pandemic. The educators, however, showed vulnerability and they shared their humanity with us, trying to build connections and relationships with students, calling us by name, utilising breakout rooms to get to know us, constantly available to us both over Zoom and email. I don’t think I knew how lucky I was, until invited back the university community for an event and it felt like returning home. I’m aware other students did not have this experience and I’m aware many educators could not offer this experience.*

*I mention this because I believe it has helped me to understand the struggles educators have faced over the pandemic; I know that they are human and they are trying their best.. I know I brought kindness, compassion and understanding to this focus group today. As we laughed introducing ourselves, and forged connections with one another, I could feel that the rapport built in these first few minutes would make a difference in the quality of data collected, as we felt comfortable chatting and joking with one another. I feared there was one participant who may have felt intimidated by the more senior and chatty staff members. I wondered if she felt less confident in her opinion, or less confident interjecting. I made sure to ask this participant, ‘what do you think?’ as the discussion wound down, before moving onto the next question, and this gave them a chance to have their voice heard.*

Data collection day #3

*Coming from a background in psychology, with extensive research training and awareness for individual differences and perspectives, I believe I bring a unique perspective to this role. I’m aware that my role is to explore the participants’ feelings, thoughts, attitudes, and experiences towards a research topic. I’m not expected to know everything when I’m in these interviewers; it is the participant who is the expert in their lived experiences. This brings me a feeling of calmness, and I feel more comfortable asking participants, ‘could you tell me what you mean when you say…’.*

*This morning, when a participant was recounting their experience of an online OSCE, they took for granted that this is assessment that I am familiar with, as I am not from a medical background. When the participant paused, I was not afraid to say, ‘I’m sorry, but could you please tell me more about the process of an online OSCE? It’s something I’m not familiar with’. I think this was perceived by the student as a moment of vulnerability for me, and gave them a feeling of power in their knowledge and expertise. It felt good to combat the possible power imbalance that can present between a researcher and a participant. When they explained the process of an online OSCE to me, I could prompt them for some clarity around why they felt this assessment was identical to an in-person OSCE. This brought greater depth and richness to the data collected and it helped me to understand my participants experience and feelings better. The knowledge and understanding I gained in this moment of vulnerability will help me to approach data analysis of this transcript and others with greater understanding.*
